# Zebrafish as a Model Organism for Research in Rare Genetic Neuromuscular Diseases

**DOI:** 10.3390/ijms26188832

**Published:** 2025-09-10

**Authors:** Eylem Emek Akyürek, Martina Erba, Francesco Dalla Barba, Dorianna Sandonà, Roberta Sacchetto

**Affiliations:** 1 Department of Comparative Biomedicine and Food Science, University of Padova, Viale dell’Università 16, Legnaro, 35020 Padova, Italy; eylememek.akyurek@unipd.it (E.E.A.); martina.erba@unipd.it (M.E.); 2 Department of Biomedical Sciences, University of Padova, 35131 Padova, Italy; francesco.dallabarba@unipd.it

**Keywords:** brody myopathy, *Danio rerio*, duchenne muscular dystrophy, limb girdle muscular dystrophies, rare neuromuscular diseases, zebrafish as disease models

## Abstract

The zebrafish (*Danio rerio*) has become one of the most popular and valuable model organisms for studying rare neuromuscular diseases. Its unique characteristics, including the high number of offspring produced with each mating, transparent eggs, rapid development, and genetic similarity to humans, make this small vertebrate ideal for investigating complex and rare disorders affecting the skeletal muscle, such as Duchenne Muscular Dystrophy (DMD), Limb Girdle Muscular Dystrophies (LGMDs), and Brody Myopathy (BM). Various zebrafish models, both natural mutants and genetically engineered strains, have been developed to study these conditions. These models enable the deciphering of pathogenetic mechanisms, the real-time monitoring of disease progression, high-throughput drug screening, and the testing of novel therapeutic approaches. As research progresses, zebrafish models are likely to play an increasingly crucial role in unravelling the complexities of rare neuromuscular diseases and developing targeted therapies, offering hope for affected patients.

## 1. Introduction

The zebrafish (*Danio rerio*) has emerged as a valuable model organism in biomedical research, particularly in the study of rare neuromuscular diseases. This small freshwater teleost, native to South Asian rivers, derives its name from the distinctive horizontal blue stripes adorning its body. Adult zebrafish, measuring approximately 2.5 cm in length, reach sexual maturity within 3–4 months post-fertilization and can produce hundreds of eggs at weekly intervals [1]. Several characteristics make zebrafish an attractive model for scientific investigation. Over 70% of human proteins, including approximately 82% of those associated with disease, have an ortholog in zebrafish [2]. The transparency of embryos and larvae allows for the direct observation of fish development. At the same time, their cost-effective maintenance and small space requirements for adults offer advantages over traditional animal models, such as rodents. Furthermore, as the fertilization of the eggs is external and occurs in aquatic environments, both embryos and zebrafish larvae are readily accessible for manipulation and observation throughout all developmental stages [1]. Additionally, researchers can employ various techniques with zebrafish, including, among others, fluorescent tracer time-lapse lineage analysis, as well as single-cell transplantation, thereby enhancing the depth and diversity of investigations. The zebrafish model has proven valuable in developmental biology [3], neurobiology [4,5,6], virology [7,8], microbiology [9,10,11], genetics [12], and drug research. A key feature of zebrafish is its immune system, which encompasses both innate and adaptive components, mirroring the mammalian immune system [13,14]. Zebrafish have been extensively used as models to replicate various human disorders due to their physiological and genetic similarities to humans. For instance, zebrafish have been employed to study congenital heart defects, as they share key characteristics with the human cardiovascular system, including heart rate, excitation patterns, myocyte action potential, morphology, and ion channel function [15,16,17,18,19]. In addition, the zebrafish has served as an effective model for investigating cancers [20,21], neurological disorders [22,23], liver dysfunction [24], kidney disease [25], blood disorders [26], and neuromuscular disorders [27,28,29]. Notably, the structural and molecular similarity between the zebrafish and human neuromuscular systems make this fish a uniquely valuable model for studying a wide range of human neuromuscular disorders (NMDs).

### 1.1. Zebrafish Neuromuscular System Organization

Zebrafish spinal motor neurons (MNs) are divided into two groups based on their innervation of target muscle fibers: primary motor neurons (PMNs) and secondary motor neurons (SMNs). PMNs consist of three subtypes. These include (1) caudal PMNs (CaP) innervating ventral trunk musculature, (2) middle PMNs (MiP) innervating dorsal trunk musculature, and (3) rostral PMNs innervating the remaining muscle fibers between ventral and dorsal trunk musculatures [29,30]. SMNs are located more ventrally than PMNs in the motor column and consist of two different types. The dorso-ventrally projecting SMNs (dvS) innervate both dorsal and ventral musculature, and the ventrally projecting SMNs (vS) have ventrally restricted arborization fields. These two types of SMNs exhibit extensive branching deep into the muscle fibers from the main axonal branch, indicating that they preferentially activate fast muscles. The dvS innervate the dorsal musculature and activate fast skeletal muscles [29].

**Figure 1:**
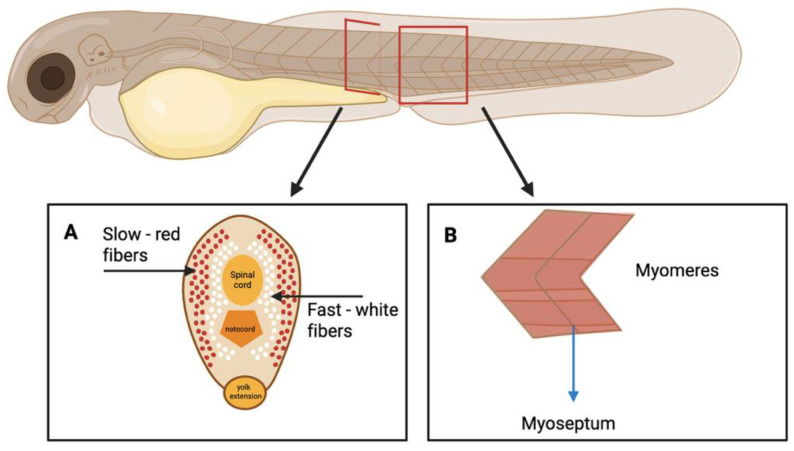
Zebrafish muscle organization. Created in BioRender. Akyurek, E.E. (2025) https://BioRender.com/o9ebmof. (**A**) Schematic cross-section of muscle fiber types placement in zebrafish body. (**B**) Schematic description of myomeres, small functional units of skeletal muscle separated by myosepta.

In zebrafish, slow-twitch skeletal muscles are located beneath the skin, while fast-twitch muscles are situated more internally (Figure 1A) [31,32,33]. Slow-twitch fibers, primarily used for sustained and continuous swimming, are found in a narrow V-shaped region adjacent to the lateral line. These fibers are characterized by their small diameter, high mitochondria and glycogen content, and aerobic metabolism. In contrast, the majority of skeletal muscle in teleost fish consists of fast-twitch fibers, which have a significantly larger diameter and are specialized for rapid, high-intensity movements such as predation and escape responses [34]. Similar to mammals, zebrafish skeletal muscle fibers in larvae are segmented by myosepta into repeating structural and functional units called myomeres, which extend along the body axis (Figure 1B) [35,36].

Zebrafish and mammalian skeletal muscle fibers share numerous molecular and anatomical features, including myofibrillar organization, contractile properties, and distinct muscle fiber types (fast- and slow-twitch) [37]. These similarities have established zebrafish as a powerful model for studying human neuromuscular diseases, further supported by the availability of advanced genome editing techniques that enhance the precision and scope of zebrafish-based research.

### 1.2. Zebrafish Genome Editing

In the early 1980s, Streisinger and colleagues pioneered the use of zebrafish as a model organism for genetic studies (3). Since then, gene editing technology in zebrafish has significantly advanced. Initial approaches involved gene knockdown using antisense morpholino oligonucleotides. These were followed by the introduction of transgenesis techniques such as the Tol2 transposon system for DNA insertion into the genome [38]. These developments ultimately led to the adoption of powerful genome editing tools, including CRISPR/Cas9, which now enable precise gene knockout and knock-in strategies that are widely used in functional genomics studies [39,40,41].

Genome editing methods have evolved over the last three decades. In the field of targeted nucleases and their possible application, four fundamental mechanisms for site-specific genome editing have emerged; together, these form a basis for new medical and agricultural advances. These four mechanisms involve meganucleases (MegNs), zinc finger nucleases (ZFNs), transcription activator-like effector nuclease (TALENs), and clustered regularly interspaced short palindromic repeats (CRISPR)/CRISPR-associated protein 9 (Cas9) (CRISPR/Cas-9) [42].

These nuclease systems can be generally categorized into two groups depending on their mechanism of DNA recognition. First, ZFNs, TALENs, and meganucleases achieve precise DNA binding through interactions between proteins and DNA; second, Cas9 is directed to specific DNA sequences through a short RNA guide molecule that forms direct base pairs with the target DNA and through interactions between proteins and DNA [43]. Targeted genome engineering, involving the modification of specific loci in diverse cell types or entire organisms, has evolved into a potent and widely applicable tool in biomedical research [44].

The convergence of advanced genetic manipulation methods and the biological parallels between zebrafish and humans have reinforced the zebrafish’s role as a prominent and valuable vertebrate model for human diseases.

## 2. Rare Neuromuscular Disease

Rare diseases are life-threatening or chronically debilitating conditions affecting fewer than 1 in 2000 individuals, as considered by the European Union. (Rare disease—European Commission. https://health.ec.europa.eu/rare-diseases-and-european-reference-networks/rare-diseases_en#:~:text=Low%20prevalence%20means%20rare%20diseases,Council%20of%2029%20April%201999 accessed on 8 September 2025) Genetic neuromuscular diseases (NMDs) encompass a group of uncommon inherited disorders affecting the function of peripheral nerves or muscles or the neuromuscular junction. The study of rare neuromuscular disorders (NMDs) faces significant challenges due to their high heterogeneity and low prevalence [45]. Drug screening for these conditions is often costly and limited by translational gaps between laboratory research and clinical application. Zebrafish have emerged as a valuable preclinical model in this context, thanks to their genetic and physiological similarities to humans or transgenic technologies [46].

Approximately 90% of neuromuscular diseases (NMDs) are classified as rare disorders [47]. Given the broad spectrum of rare NMDs, this review focuses on the use of zebrafish models to study a selection of specific rare and ultra-rare conditions, namely (1) Duchenne Muscular Dystrophy, (2) Limb Girdle Muscular Dystrophies (LGMDs), and (3) Brody Myopathy. These disorders are caused by mutations in different genes encoding essential proteins involved in muscle structure and function. For each disease, we will examine how zebrafish models have advanced our understanding of the underlying pathology and contributed to the development of potential therapeutic strategies.

### 2.1. Duchenne Muscular Dystrophy (DMD)

Duchenne Muscular Dystrophy is the most common muscular dystrophy, with a prevalence of 1 in every 5000 males [48]. The primary cause of DMD is mutations in the X-linked DMD gene, located at Xp21.2-p21.1, which encodes the dystrophin protein [49]. DMD is characterized by progressive muscle dysfunction, beginning with motor development deficiency in infants. It progresses to the loss of ambulation by the end of the first decade of life and ultimately leads to respiratory or cardiac failure in early adulthood. The disease is usually fatal in the third/fourth decade due to these complications [50]. Currently, corticosteroids are the gold-standard treatment to delay the progression of the muscle dysfunction, even though secondary effects must be carefully monitored [51,52]. Furthermore, exon skipping approaches have also been approved for some specific DMD mutations, and gene therapy strategies using mini- and micro-dystrophin are under investigation [53]. There are over 60 laboratory animals, ranging from small invertebrates like flies and nematodes to mammals such as mice and canines with dystrophin deficiency [54]. While the need to find new therapeutic strategies is ever present, having reliable preclinical models could support the discovery of novel treatments for DMD. In this scenario, zebrafish offer many advantages. Their skeletal muscle phenotypes can be readily observed using the birefringence technique, allowing for the phenotype-based screening of small molecules, thus avoiding the need for premature suppression of chemically treated specimens during ongoing studies. Furthermore, their small size allows for the easy permeation of chemical compounds, and their possibility to be studied in large quantities makes zebrafish ideal for high-throughput screening processes [55,56,57].

Zebrafish is currently the most widely used model of DMD, both to investigate the disease’s pathological mechanism and to enable high-throughput and high-content drug screening. Five dystrophin-null zebrafish models of DMD have been identified through genetic screening after ethylnitrosourea (ENU) mutagenesis [58,59]. Four zebrafish lines, sapje (*dmd^ta222a^*), *dmd^pc1^*, *dmd^pc2^*, and *dmd^tm90c^*, have been identified as carrying nonsense mutations in exon 4, exon 21, exon 32, and exon 53 of the *dmd* gene, respectively [59,60,61]. The sapje-like (sap^cl100^) model contains a mutation at the donor splice site of exon 62 [59]. More recently, these models have been used in molecular library drug screening and drug repurposing studies to discern whether candidate drugs were capable of correcting the skeletal muscle phenotype [51,55,62,63,64,65].

In addition to these models, with gene knockout or knockdown technologies new zebrafish models were generated. In Nesari et al. [66], *jagged1* overexpression was induced in dystrophin-knockout zebrafish larvae by co-injecting a Jagged1 expression plasmid into embryos lacking *dmd*.

In a recent study aiming to identify genetic factors that influence the severity of DMD, researchers developed a CRISPR/Cas9 screening pipeline. As a validation step, they knocked down homologs of known DMD modifiers (LTBP4 and THBS1) and observed effects on disease severity in dmd^−/−^ zebrafish. They then identified and validated *galntl6*, *man1a1*, *etaa1a*, *etaa1b*, and *adamts17* as bona fide DMD modifiers in this model [67]. Zebrafish represent an exceptionally suitable model organism for phenotype-based chemical screening in the identification of potential therapeutic compounds. Their utility may also extend to drug screening for DMD mutations [64], which are still orphan diseases, offering new opportunities to identify agents capable of ameliorating muscle-related pathologies.

### 2.2. Limb Girdle Muscular Dystrophies (LGMDs)

LGMDs are a family of heterogeneous disorders, characterized by the involvement of the proximal musculature of the pelvic and shoulder girdles [68]. LGMDs are different from the X-linked DMD/BMD as they affect both males and females and show either autosomal dominant (LGMD D) or autosomal recessive (LGMD R) inheritance [69]. The clinical manifestation may be variable, ranging from severe forms, with rapid onset and progression, characterized by a Duchenne-like muscular phenotype, to very mild forms, allowing affected people to have an almost normal life span and physical activity [70,71,72]. As mentioned before, LGMDs are a family of heterogeneous disorders and the classification of the LGMD types is reported in Table 1.

Currently, there is no cure for LGMDs, and only corticosteroids (used on the basis of the DMD readout), physical therapy, and/or supportive treatment are used to manage symptoms and increase the quality of life of patients [143,144]. Calpainopathy and dysferlinopathy are the most frequent LGMD subtypes. Calpainopathy (LGMDR1) is an autosomal recessive dystrophy characterized by symmetric and progressive weakness of proximal limb girdle muscles. The *CAPN3* gene is responsible for this type of muscular dystrophy [86]. To date, several homozygous or compound heterozygous CAPN3 mutations have been linked with LGMDR1 [88,145]. Based on the distribution of muscle weakness and age onset, calpainopathy was divided into three different groups [146].

To better understand the molecular mechanism of calpainopathies, Prykhozhij et al. [87] generated *capn3b*-inactivated zebrafish models with CRISPR/Cas9 technology to study its mutant phenotype as a potential LGMDR1 disease model. They detected muscle damage in these fish under challenging conditions and they concluded that this model might be valuable for the evaluation of whole-organism therapeutic interventions and behavioral phenotyping in the context of calpainopathies [87,88,89,90,91,92,93,94,95,96,97,98,99,100,101,102,103,104,105,106,107,108,109,110,111,112,113,114,115,116,117,118,119,120,121,122,123,124,125,126,127,128,129,130,131,132,133,134,135,136,137,138,139,140,141,142,143,144,145,146,147].

Dysferlinopathy (LMGDR2) is one of the autosomal recessive LGMDs, caused by a homozygous or compound heterozygous mutation in the *DYSF* gene encoding dysferlin, a protein involved in sarcolemma repair. The disease typically manifests between ages 13 and 40 with initial proximal thigh muscle weakness, sparing the shoulder muscles initially. A distinctive walking style, known as the “dysferlin gait,” is considered a characteristic feature among patients with LGMDR2 [148]. The morpholino knockdown zebrafish model was used to understand the underlying mechanism of dysferlinopathy [90] and the dysferlin protein’s role in the stabilization of muscle structure and sarcolemma repair [91].

Among LGMDRs are four distinct MD subtypes, called sarcoglycanopathies. Sarcoglycanopathies are characterized by the loss or strong reduction in sarcoglycans at the sarcolemma [149,150]. Sarcoglycanopathies are caused by mutations in the *SGCA*, *SGCB*, *SGCG*, and *SGCD* genes that code for α-, β-, γ-, and δ-sarcoglycan (SG), respectively.

Assessing the prevalence of sarcoglycanopathy is challenging, partly due to its association with specific ethnic groups and geographical regions. Nevertheless, it accounts for 10–20% of all LGMD cases where dystrophin levels are normal [151,152]. Among the sarcoglycanopathy subtypes, LGMDR3 is the most commonly reported, followed by LGMDR5 and LGMDR4, while LGMDR6 is the least frequently observed. The need to find a therapeutic solution for sarcoglycanopathies, which are still orphan diseases, prompts researchers to use different animal models. Several rodent models of sarcoglycanopathy are available, both naturally occurring and resulting from genome modification [153]. Significant efforts are ongoing to develop new cures. In the last few years, gene therapy has reached Phase 3 clinical trials for LGMDR4 and the end of the trial will allow for the evaluation of its safety and efficacy [154].

In addition to gene therapy, several other approaches are under investigation. These include cell-based therapies, which aim to repair or regenerate damaged muscle fibers without causing any immunologic reaction [155]. Also, small-molecule-based treatment with CFTR (Cystic Fibrosis Transmembrane Regulator) correctors has shown efficacy in restoring sarcoglycan complex integrity and improving muscle pathology in preclinical models of LGMDR3 [156,157,158].

Since the zebrafish is gaining increased attention as an animal model of human diseases, sarcoglycanopathies have been modelled in this small vertebrate. A severe phenotype was developed by knocking down either β- or δ-SG through the use of antisense morpholino oligonucleotides directed toward the starting codon of both *sgcb* and *sgcd* mRNA [100,101]. More recently, δ- and β-SG knockout zebrafish lines have been generated by applying the CRISPR/Cas9 genome editing technique by Dalla Barba et al. [96]. The in-depth characterization of these two novel zebrafish mutants highlighted the progressive development of the dystrophic phenotype, strictly mirroring the human condition. These diverse animal models, ranging from rodents to zebrafish, provide valuable tools for understanding the pathophysiology of sarcoglycanopathies and testing potential therapeutic interventions.

The literature currently presents a limited number of zebrafish models for these rare diseases. The morpholino knockdown technique has emerged as a predominant method for LGMD zebrafish research. For instance, a knockdown hnrpdl morpholino zebrafish model was used to establish the association between HNRPDL and LGMDD3. These zebrafish exhibited body shape abnormalities, tail twisting, restricted mobility, and uncoordinated movements, consistent with myopathic manifestations [79]. Zhang et al. [104] employed a similar approach to investigate tcap mutation pathogenesis in LGMDR7, showing that it is associated with a disruption of sarcomere–T–tubular interaction, but not of sarcomere assembly per se.

In addition, different knockdown fkrp models [109,110,111,112] were studied to understand Walker–Warburg syndrome (WWS), LGMDR9, and congenital muscular dystrophy 1C (MDC1C), which share different mutations in the FKRP gene. Recent developments in genome editing have led to the creation of fkrp zebrafish mutants using TALENs and heat shock-inducible FKRP-transgenic zebrafish using a Tol2-based-transgenesis system as a novel model for LGMDR9 [107]. Additionally, other fkrp zebrafish mutants were generated with zinc finger nucleases and CRISPR/Cas9. These mutants provided new information on the role of fkrp. It revealed that fkrp is involved in the sialylation of fibronectin in the Golgi apparatus, a process crucial for fibronectin–collagen binding and maintaining the integrity of the muscle basement membrane [108]. FKRP gene mutations have also been involved in α-Dystroglycanopathies together with PMOT1 (LGMDR11), FKTN (LGMDR13), PMOT2 (LGMDR14), and POMGNT1 (LGMDR15) gene mutations. There are several zebrafish models that can support the study of the α-Dystroglycan complex (distinct from the LGMD context, which will not be mentioned in this study).

Since different mutations in the same gene can cause different diseases, there are several studies where new zebrafish models were generated in the *ttn* (LGMDR10) [115,116], *dag1* (LGMDR16) [126,127], and *lama2* (LGMDR23) [136,137] genes. These mostly showed phenotypes related to other myopathies or disorders.

As stated above, the morpholino knockdown model is one of the most used animal models in LGMDs; the model helps highlight the effects of mutated proteins on muscle development and phenotype. With trappc11 knockdown morphants, Ulhaq et al. [130] demonstrated the effects of FGF8 (Fibroblast Growth Factor 8) in pathogenesis and its therapeutic potential for LGMDR18. The knockdown of zebrafish GMPPB demonstrated a similar phenotype to that in humans and revealed new insights about α-DG glycosylation in LGMDR19 [131]. Some LGMD types contain mutations in the same gene, but these are based on inheritance, and they can differ, like the LGMDD5 and LGMDR22 cases. Different techniques are used to highlight the pathogenetic mechanism of cal6a mutations, the main cause of LGMDD5 and LGMDR22. The morpholino knockdown method used to understand the role of the cal6a mutation and exon 13 knockdown in col6a1 resulted in a similar human myopathic phenotype in zebrafish [85]. The TALEN Exon Skipping design was used to generate col6^a1ama605003^ mutations in exon 14; this yielded a mild and progressive phenotype with which to study LGMDD5 [84]. With the CRISPR/cas9-mediated genome editing method, col6a1-null zebrafish were generated by Tonelotto et al. [83], revealing that COL6 plays a crucial role during development.

Similarly, a popdc1 knockdown model in zebrafish revealed myofibrillar misalignment, aberrant myotendinous junction formation, myofiber detachment, and reduced membrane localization of Popdc1 and Popdc2 in the skeletal muscle of homozygous popdc1 mutants. The observed phenotype resembled that of LGMDR25 patients, suggesting that popdc1 mutations result in a mild, late-onset LGMDs [139]. In the case of popdc3, knockdown zebrafish displayed curved tails and dystrophic alterations in muscle tissues, including muscle fiber detachment and disarray. These findings led to the identification of a novel autosomal recessive LGMD subtype (LGMDR26) [140].

Beyond the morpholino knockdown technique, researchers have employed the injection of RNA encoding mutant human DNAJB6 into zebrafish embryos to elucidate the mechanisms underlying muscle defects caused by DNAJB6 mutations in LGMDD1 patients [75,76].

It is noteworthy that, in addition to these zebrafish model approaches, numerous murine models have been developed for various LGMD subtypes, further expanding the research tools in this field and increasing the possibility of performing library drug screening or drug repurposing research.

### 2.3. Brody Myopathy (BM)

BM is an ultra-rare, autosomal recessive inherited myopathy, caused by homozygous or compound heterozygous mutations in the ATP2A1 gene encoding the SERCA protein, isoform 1. It is localized on the 16p11 chromosome [159].

In 1969, Brody first described in a patient a muscular disorder characterized by an “exercise-induced impairment of muscle relaxation” [160]. Through experiments of Ca uptake using isolated sarcoplasmic reticulum (SR) vesicles, Brody reported reduced Ca^2+^ transport activity. Because Sarco/Endoplasmic Reticulum Ca^2+^-ATPase (SERCA) plays a critical role in muscle relaxation, he concluded that a decrease in Ca^2+^ uptake would be the cause of the human pathology. Typical and common symptoms of BM include muscle exercise-induced stiffness and difficulty in relaxing skeletal muscles following contracture. Patients experience difficulties with basic and essential exercises, such as running and climbing stairs. Stiffness develops within a few minutes after exercise is started, preventing the patients from using their muscles. After a short period of rest, relaxation occurs and exercise is possible again. The symptoms begin in childhood and the course of the disease is stationary or slowly progressive. Exercise-induced skeletal muscle delay relaxation is most pronounced in the legs, arms, and eyelids [160,161,162,163,164,165]. Additionally, after exposure to cold temperatures myalgia and muscle cramps may worsen [159].

Currently, no specific pharmacological therapy exists for BM. Certain muscle relaxants, such as Dantrolene, or calcium channel blockers, like Verapamil, have demonstrated effectiveness in relaxing muscle stiffness and enhancing exercise tolerance; however, they remain merely symptomatic treatments [162,165,166]. The attempt to generate a mouse model for BM by knocking out the SERCA1 gene had unexpected consequences. While it did cause limb stiffness, it also severely impaired diaphragm function. This led to new-born mice dying from respiratory failure within just two hours [167]. Given these challenges with mouse models, researchers sought out a different animal model. Recently, bovine congenital pseudomyotonia (PMT) was recognized as a unique mammalian model for BM [168,169,170]. On the other hand, zebrafish was proposed as a non-mammalian vertebrate model of BM; this proposal was made almost simultaneously in 2004 by both Hirata et al. [171] and Gleason et al. [172]. These authors named the zebrafish mutant lines, generated by the Tūbingen mutagenesis screen [58], as “Accordion (acc)” in analogous reference to the action of the musical instrument, since mutants fail to coil their tails normally but contract the bilateral trunk muscles simultaneously to shorten the trunk, just like an accordion. The acc mutant lines present three different mutations: acc^mi25i^, acc^mi289a^, and acc^tq206^, identified in the atp2a1 gene encoding SERCA1 in zebrafish.

Specifically, acc^tq206^, carrying a point mutation at position 2297, presented the strongest phenotype with a serine (Ser) replaced with phenylalanine (Phe) at position 766 (S766F) of the SERCA1 protein.

SERCA1 comprises a large cytoplasmic headpiece, short luminal loops, and a transmembrane domain made of 10 alpha-helices, named M1-M1 [173]. The mutation S766F is located in the portion of the M5 domain called the “flexible hinge” (from amino acid Ile765 to Asn768). This region is highly conserved in mammals as well as in zebrafish. A conformational change occurred from the Ca^2+^ binding to the dissociation state; this demonstrated that the middle part of the M5 domain of the SERCA protein rotates approximately 30° during these events, confirming the functional role of the flexible hinge. As previously stated by Hirata et al. [171] and Gleason et al. [172], the substitution of the polar side chain of Ser with the non-polar side chain of Phe at position 766 of the M5 hinge region is expected to restrict the conformational freedom of the protein. As a result, this mutation at the M5 domain could disrupt or severely reduce SERCA1 function, reinforcing the essential role of the M5 domain. These events could explain the extremely severe phenotype found in acc^tq206^ [174]. The acc^tq206^ mutant has been used to test a potential innovative pharmacological therapy to cure Brody patients based on CFTR correctors. The results obtained, although preliminary, demonstrated that this line represents a valuable model in assessing drug tolerability, as no significant toxic effect was observed in the accordion larvae upon CFTR corrector treatment.

## 3. Conclusions

Cell culture technique and the most recent development of induced pluripotent stem cell (iPSC) sophisticated technology represent fundamental tools for assessing experimental settings, understanding human disease mechanisms, and testing therapeutic hypotheses. Moreover, in the last decade these in vitro models have been improved by the three-dimensional cell culture (3D) technique. This in vitro cellular model exhibits an enhanced capacity to recapitulate the structural organization and microscopic architecture of a living organ [175].

However, in vitro studies, albeit fundamental, are often the first step toward a cure. Preclinical investigations, involving in vivo animal studies, are essential in drug development before a clinical trial can be pursued.

The zebrafish model has been widely adopted in both animal and human health research, and, more recently, in aquaculture. Although rodents remain the most commonly used model globally, the use of zebrafish has grown significantly within the scientific community in the past few decades. Various factors contributed to this significant growth, based on a comparison of the advantages and disadvantages (Table 2) of zebrafish and rodent models and their utility for the chosen study area.

Notably, employing the zebrafish model leads to a reduction in time and resource utilization compared to murine models. In addition, it provides more informative and predictive data than in vitro systems. As a result, zebrafish contribute to the replacement of and reduction in mammalian use in research, aligning with ethical animal welfare preferences. Furthermore, zebrafish can serve as a valuable confirmatory model to validate previously obtained positive results, thereby enhancing the robustness and reproducibility of scientific findings [185].

The zebrafish has become an invaluable model organism in biomedical research, especially for studying rare neuromuscular diseases. The zebrafish’s unique features, such as rapid development, transparent embryos, and strong genetic similarity to humans, make it particularly well suited for investigating complex conditions like DMD, LGMDs, and BM.

The generation of zebrafish models, whether through natural mutations or advanced genetic engineering, has greatly enhanced our understanding of these disorders. They enable the real-time observation of disease progression and are highly effective for high-throughput drug screening, as well as the testing of novel therapeutic strategies. For DMD, models like sapje and sapje-like mutants have been instrumental in clarifying the mechanism of dystrophin deficiency and assessing potential treatments. In LGMD research, zebrafish models have provided critical insights into disease biology and supported large-scale, phenotype-based drug discovery efforts. In contrast, BM still relies primarily on the Acc zebrafish line as the only widely available animal model for studying disease mechanisms and evaluating therapeutic options.

The growing use of zebrafish in these areas highlights the model’s versatility and value in bridging the gap between basic research and preclinical applications. As research progresses, zebrafish models are poised to play an increasingly central role in uncovering the complexities of rare neuromuscular diseases and in advancing the development of targeted, effective therapies. This progress not only deepens scientific knowledge but also brings new hope to patients affected by these challenging conditions.

## Figures and Tables

**Table 1:** Classification of Limb Girdle Muscular Dystrophy types. AD: autosomal dominant, AR: autosomal recessive.

Inheritance	Location	Gene	Phenotype	Abbreviation	Reference for Gene	Zebrafish Model	Reference For ZF
AD	7q36.3	DNAJB6	DNAJB6-related muscular dystrophy	LGMDD1	[73,74]	MO mRNA injection	[75,76]
AD	7q32.1	TNPO3	TNP03-related muscular dystrophy	LGMDD2	[77,78]	-	-
AD	4q21.22	HNRNPDL	HNRNPDL-related muscular dystrophy	LGMDD3	[79]	MO	[79]
AD	15q15.1	CAPN3	Calpain3-related muscular dystrophy	LGMDD4	[80,81]	-	-
AD	21q22.3	COL6A	Bethlem myopathy	LGMDD5	[82]	CRISPR/Cas9 TALEN MO	[83,84,85]
AR	15q15.1	CAPN3	Calpainopathies	LGMDR1	[86]	CRISPR/Cas9 TALEN	[87,88]
AR	2p13.2	DYSF	Dysferlinopathy	LGMDR2	[89]	MO	[90,91]
AR	17q21.33	SGCA	α-sarcoglycanopathy	LGMDR3	[92,93]	-	-
AR	4q12	SGCB	β-sarcoglycanopathy	LGMDR4	[94,95]	CRISPR/Cas9	[96]
AR	13q12.12	SGCG	γ-sarcoglycanopathy	LGMDR5	[97,98]	-	-
AR	5q33.2-q33.3	SGCD	δ-sarcoglycanopathy	LGMDR6	[99]	CRISPR/Cas9 MO	[96,100,101]
AR	17q12	TCAP	Telethonin-related muscular dystrophy	LGMDR7	[102,103]	MO	[104]
AR	9q33.1	TRIM32	Sarcotubular myopathy	LGMDR8	[105]	-	-
AR	19q13.32	FKRP	FKRP related muscular dystrophy	LGMDR9	[106]	TALEN, Tol2 transposon system, CRISPR/Cas9, MO	[107,108,109,110,111,112]
AR	2q31.2	TTN	Tinin-related muscular dystrophy	LGMDR10	[113,114]	CRISPR/Cas9 MO	[115,116]
AR	9q34.13	POMT1	POMT1-related muscular dystrophy	LGMDR11	[117,118]	MO	[119]
AR	11p14.3	ANO5	Anoctamin5-related muscular dystrophy	LGMDR12	[120]	-	-
AR	9q31.2	FKTN	Fucutin-related muscular dystrophy	LGMDR13	[121]	-	-
AR	14q24.3	POMT2	POMT2-related muscular dystrophy	LGMDR14	[122]	MO	[119]
AR	1p34.1	POMGnT1	POMGnT1-related muscular dystrophy	LGMDR15	[123,124]	-	-
AR	3p21.31	DAG1	α-dystroglycan-related muscular dystrophy	LGMDR16	[117,125]	MO ENU screening	[126,127]
AR	8q24.3	PLEC1	Plectin-related muscular dystrophy	LGMDR17	[128]	-	-
AR	4q35.1	TRAPPC11	TRAPPC11-related muscular dystrophy	LGMDR18	[129]	MO	[130]
AR	3p21.31	GMPPB	GMPPB-related muscular dystrophy	LGMDR19	[131]	MO	[131]
AR	7p21.2	ISPD (CRPPA)	ISPD-related muscular dystrophy	LGMDR20	[132]	-	-
AR	3q13.33	POGLUT1	POGLUT1-related muscular dystrophy	LGMDR21	[133]	-	-
AR	21q22.3	COL6A	Autosomal recessive Bethlem myopathy	LGMDR22	[134]	CRISPR/Cas9 TALEN MO	[83,84,85]
AR	6q22.33	LAMA2	Laminin α2-related muscular dystrophy	LGMDR23	[135]	MO ENU screening	[136,137]
AR	3p22.1	POMGNT2	PMGNT2-related muscular dystrophy	LGMDR24	[138]	-	-
AR	6q21	BVES (POPDC1)	Muscular dystrophy	LGMDR25	[139]	MO	[139]
AR	6q21	POPDC3	Muscular dystrophy	LGMDR26	[140]	MO	[140]
AR	14q32.33	JAG2	Muscular dystrophy	LGMDR27	[141]	-	-
AR	5q13.3	HMGCR	Muscular dystrophy	LGMDR28	[142]	-	-

**Table 2:** Comparison of zebrafish and rodent models for studying rare neuromuscular diseases.

Zebrafish	Rodents
70% of homologous gene conservation with humans [2]	85% of homologous gene conservation with humans [2]
Maintenance cost is low [176]	Maintenance cost is high [176]
Possibility of genetic modification [175]	Possibility of genetic modification [175]
Rapid development and transparency of eggs [1]	Development takes time [176]
Suitable for embryonic developmental studies [1]	Embryonic studies require maternal sacrifice [176]
Rapid phenotyping [176]	Slow phenotyping [176]
Back-crossing is rapid compared to mice [176]	Back-crossing is slower compared to zebrafish [176]
Genes, molecular pathways, and organ systems are conserved with humans’ less than those of mice [176]	Genes, molecular pathways, and organ systems are highly conserved with humans’ [176]
Compound screening is easy [176,177,178,179,180]	Compound screening is laborious
High number of offspring [1,2,3,4,5,6,7,8,9,10,11,12,13,14,15,16,17,18,19,20,21,22,23,24,25,26,27,28,29,30,31,32,33,34,35,36,37,38,39,40,41,42,43,44,45,46,47,48,49,50,51,52,53,54,55,56,57,58,59,60,61,62,63,64,65,66,67,68,69,70,71,72,73,74,75,76,77,78,79,80,81,82,83,84,85,86,87,88,89,90,91,92,93,94,95,96,97,98,99,100,101,102,103,104,105,106,107,108,109,110,111,112,113,114,115,116,117,118,119,120,121,122,123,124,125,126,127,128,129,130,131,132,133,134,135,136,137,138,139,140,141,142,143,144,145,146,147,148,149,150,151,152,153,154,155,156,157,158,159,160,161,162,163,164,165,166,167,168,169,170,171,172,173,174,175,176]	Small number of offspring [176]
The permeability of embryos facilitates compound screening in fish water [176]	In some cases, mouse models do not mimic human phenotype [167,177,178,179]
Low experimental reagent availability [180]	High experimental reagent availability [180]
Less strict regulations for zebrafish use until 5dpf [181,182,183,184]	Very strict regulations [183,184]

## References

[1] Kimmel C.B., Ballard W.W., Kimmel S.R., Ullmann B., Schilling T.F. (1995). Stages of Embryonic Development of the Zebrafish. Dev. Dyn..

[2] Howe K., Clark M.D., Torroja C.F., Torrance J., Berthelot C., Muffato M., Teucke M. (2013). The Zebrafish Reference Genome Sequence and Its Relationship to the Human Genome. Nature.

[3] Streisinger G., Walker C., Dower N., Knauber D., Singer F. (1981). Production of Clones of Homozygous Diploid Zebra Fish (Brachydanio Rerio). Nature.

[4] Haffter P., Odenthal J., Mullins M.C., Lin S., Farrell M.J., Vogelsang E., Haas F., Brand M., van Eeden F.J., Furutani-Seiki M. (1996). Mutations Affecting Pigmentation and Shape of the Adult Zebrafish. Dev. Genes Evol..

[5] Panula P., Sallinen V., Sundvik M., Kolehmainen J., Torkko V., Tiittula A., Moshnyakov M., Podlasz P. (2006). Modulatory Neurotransmitter Systems and Behavior: Towards Zebrafish Models of Neurodegenerative Diseases. Zebrafish.

[6] Chandrasekhar A., Guo S., Masai I., Nicolson T., Wu C.F. (2017). Zebrafish: From Genes and Neurons to Circuits, Behavior and Disease. J. Neurogenet..

[7] Phelan P.E., Pressley M.E., Witten P.E., Mellon M.T., Blake S., Kim C.H. (2005). Characterization of Snakehead Rhabdovirus Infection in Zebrafish (Danio Rerio). J. Virol..

[8] Ludwig M., Palha N., Torhy C., Briolat V., Colucci-Guyon E., Bremont M., Herbomel P., Boudinot P., Levraud J.P. (2011). Whole-Body Analysis of a Viral Infection: Vascular Endothelium Is a Primary Target of Infectious Hematopoietic Necrosis Virus in Zebrafish Larvae. PLoS Pathog..

[9] Swaim L.E., Connolly L.E., Volkman H.E., Humbert O., Born D.E., Ramakrishnan L. (2006). Mycobacterium Marinum Infection of Adult Zebrafish Causes Caseating Granulomatous Tuberculosis and Is Moderated by Adaptive Immunity. Infect. Immun..

[10] Levraud J.P., Disson O., Kissa K., Bonne I., Cossart P., Herbomel P., Lecuit M. (2009). Real-Time Observation of Listeria Monocytogenes-Phagocyte Interactions in Living Zebrafish Larvae. Infect. Immun..

[11] Saralahti A., Rämet M. (2015). Zebrafish and Streptococcal Infections. Scand. J. Immunol..

[12] Petrie-Hanson L., Hohn C., Hanson L. (2009). Characterization of Rag1 Mutant Zebrafish Leukocytes. BMC Immunol..

[13] Zarkadis I.K., Mastellos D., Lambris J.D. (2001). Phylogenetic Aspects of the Complement System. Dev. Comp. Immunol..

[14] Lam S.H., Chua H.L., Gong Z., Lam T.J., Sin Y.M. (2004). Development and Maturation of the Immune System in Zebrafish, Danio Rerio: A Gene Expression Profiling, in Situ Hybridization and Immunological Study. Dev. Comp. Immunol..

[15] Zhao Y., Zhang K., Sips P., Macrae C.A. (2019). Screening Drugs for Myocardial Disease in Vivo with Zebrafish: An Expert Update. Expert Opin. Drug Discov..

[16] Genge C.E., Lin E., Lee L., Sheng X., Rayani K., Gunawan M., Tibbits G.F. (2016). The Zebrafish Heart as a Model of Mammalian Cardiac Function. Rev. Physiol. Biochem. Pharmacol..

[17] Gut P., Reischauer S., Stainier D.Y., Arnaout R. (2017). Little Fish, Big Data: Zebrafish as a Model for Cardiovascular and Metabolic Disease. Physiol. Rev..

[18] Poon K.L., Brand T. (2013). The Zebrafish Model System in Cardiovascular Research: A Tiny Fish with Mighty Prospects. Glob. Cardiol. Sci. Pract..

[19] Rafferty S.A., Quinn T.A. (2018). A Beginner’s Guide to Understanding and Implementing the Genetic Modification of Zebrafish. Prog. Biophys. Mol. Biol..

[20] Cagan R.L., Zon L.I., White R.M. (2019). Modeling Cancer with Flies and Fish. Dev. Cell.

[21] Barriuso J., Nagaraju R., Hurlstone A. (2015). Zebrafish: A New Companion for Translational Research in Oncology. Clin. Cancer Res..

[22] Kalueff A.V., Gebhardt M., Stewart A.M., Cachat J.M., Brimmer M., Chawla J.S., Craddock C., Kyzar E.J., Roth A., Landsman S. (2013). Towards a Comprehensive Catalog of Zebrafish Behavior 1.0 and Beyond. Zebrafish.

[23] Griffin A., Hamling K.R., Hong S., Anvar M., Lee L.P., Baraban S.C. (2018). Preclinical Animal Models for Dravet Syndrome: Seizure Phenotypes, Comorbidities and Drug Screening. Front. Pharmacol..

[24] Goessling W., Sadler K.C. (2015). Zebrafish: An Important Tool for Liver Disease Research. Gastroenterology.

[25] Cirio M.C., De Caestecker M.P., Hukriede N.A. (2015). Zebrafish Models of Kidney Damage and Repair. Curr. Pathobiol. Rep..

[26] Rissone A., Burgess S.M. (2018). Rare Genetic Blood Disease Modeling in Zebrafish. Front. Genet..

[27] Karuppasamy M., English K.G., Henry C.A., Manzini M.C., Parant J.M., Wright M.A., Ruparelia A.A., Currie P.D., Gupta V.A., Dowling J.J. (2024). Standardization of Zebrafish Drug Testing Parameters for Muscle Diseases. Dis. Model. Mech..

[28] Pappalardo A., Pitto L., Fiorillo C., Alice Donati M., Bruno C., Santorelli F.M. (2013). Neuromuscular Disorders in Zebrafish: State of the Art and Future Perspectives. NeuroMolecular Med..

[29] Singh J., Patten S.A. (2022). Modeling Neuromuscular Diseases in Zebrafish. Front. Mol. Neurosci..

[30] Moreno R.L., Ribera A.B. (2009). Zebrafish Motor Neuron Subtypes Differ Electrically Prior to Axonal Outgrowth. J. Neurophysiol..

[31] Bone Q. (1978). Locomotor Muscle.

[32] Devoto S.H., Melançon E., Eisen J.S., Westerfield M. (1996). Identification of Separate Slow and Fast Muscle Precursor Cells in Vivo, Prior to Somite Formation. Development.

[33] Guyon J.R., Steffen L.S., Howell M.H., Pusack T.J., Lawrence C., Kunkel L.M. (2007). Modeling Human Muscle Disease in Zebrafish. BBA-Mol. Basis Dis..

[34] Altringham J.D., Ellerby D.J. (1999). Fish Swimming: Patterns in Muscle Function. J. Exp. Biol..

[35] Stickney H.L., Barresi M.J., Devoto S.H. (2000). Somite Development in Zebrafish. Dev. Dyn..

[36] Charvet B., Malbouyres M., Pagnon-Minot A., Ruggiero F., Le Guellec D. (2011). Development of the Zebrafish Myoseptum with Emphasis on the Myotendinous Junction. Cell Tissue Res..

[37] Gibbs E.M., Horstick E.J., Dowling J.J. (2013). Swimming into Prominence: The Zebrafish as a Valuable Tool for Studying Human Myopathies and Muscular Dystrophies. FEBS J..

[38] Kawakami K., Shima A., Kawakami N. (2000). Identification of a Functional Transposase of the Tol2 Element, an Ac-like Element from the Japanese Medaka Fish, and Its Transposition in the Zebrafish Germ Lineage. Proc. Natl. Acad. Sci. USA.

[39] Klem J.R., Gray R., Lovely C.B. (2022). The Zebrafish Tol2 System: A Modular and Flexible Gateway-Based Transgenesis Approach. J. Vis. Exp..

[40] Li Y., Jia Z., Zhang S., He X. (2021). Progress in Gene-Editing Technology of Zebrafish. Biomolecules.

[41] Medishetti R., Balamurugan K., Yadavalli K., Rani R., Sevilimedu A., Challa A.K., Chatti K. (2022). CRISPR-Cas9-Induced Gene Knockout in Zebrafish. STAR Protoc..

[42] Khalil A.M. (2020). The Genome Editing Revolution. J. Genet. Eng. Biotechnol..

[43] Cox D.B.T., Platt R.J., Zhang F. (2015). Therapeutic Genome Editing: Prospects and Challenges. Nat. Med..

[44] Hsu P.D., Lander E.S., Zhang F. (2014). Development and Applications of CRISPR-Cas9 for Genome Engineering. Cell.

[45] Bhatt J.M. (2016). The Epidemiology of Neuromuscular Diseases. Neurol. Clin..

[46] Patton E.E., Zon L.I., Langenau D.M. (2021). Zebrafish Disease Models in Drug Discovery: From Preclinical Modelling to Clinical Trials. Nat. Rev. Drug Discov..

[47] Ricci G., Torri F., Bianchi F., Fontanelli L., Schirinzi E., Gualdani E., Francesconi P., Gagliardi D., Serra G., Mongini T. (2022). Frailties and Critical Issues in Neuromuscular Diseases Highlighted by SARS-CoV-2 Pandemic: How Many Patients Are Still “Invisible”?. Acta Myol..

[48] Mendell J.R., Shilling C., Leslie N.D., Flanigan K.M., al-Dahhak R., Gastier-Foster J., Weiss R.B. (2012). Evidence-based Path to Newborn Screening for Duchenne Muscular Dystrophy. Ann. Neurol..

[49] Monaco A.P., Neve R.L., Colletti-Feener C., Bertelson C.J., Kurnit D.M., Kunkel L.M. (1986). Isolation of Candidate cDNAs for Portions of the Duchenne Muscular Dystrophy Gene. Nature.

[50] Crone M., Mah J.K. (2018). Current and Emerging Therapies for Duchenne Muscular Dystrophy. Curr. Treat. Options Neurol..

[51] Farr G.H., Morris M., Gomez A., Pham T., Kilroy E., Parker E.U., Maves L. (2020). A Novel Chemical-Combination Screen in Zebrafish Identifies Epigenetic Small Molecule Candidates for the Treatment of Duchenne Muscular Dystrophy. Skelet. Muscle.

[52] Cui Y., Shao S., Zhang L., Wu J., Ma F., Cai X., Wang C. (2025). The Effects of Glucocorticoids on Cardiac Function of Patients with Duchenne Muscular Dystrophy: Benefit or Not?. Eur. J. Pediatr..

[53] Patterson G., Conner H., Groneman M., Blavo C., Parmar M.S. (2023). Duchenne Muscular Dystrophy: Current Treatment and Emerging Exon Skipping and Gene Therapy Approach. Eur. J. Pharmacol..

[54] McGreevy J.W., Hakim C.H., McIntosh M.A., Duan D. (2015). Animal Models of Duchenne Muscular Dystrophy: From Basic Mechanisms to Gene Therapy. Dis. Models Mech..

[55] Kawahara G., Karpf J.A., Myers J.A., Alexander M.S., Guyon J.R., Kunkel L.M. (2011). Drug Screening in a Zebrafish Model of Duchenne Muscular Dystrophy. Proc. Natl. Acad. Sci. USA.

[56] Lambert M.R., Spinazzola J.M., Widrick J.J., Pakula A., Conner J.R., Chin J.E., Kunkel L.M. (2021). PDE10A Inhibition Reduces the Manifestation of Pathology in DMD Zebrafish and Represses the Genetic Modifier PITPNA. Mol. Ther..

[57] Stocco A., Smolina N., Sabatelli P., Šileikytė J., Artusi E., Mouly V., Bernardi P. (2021). Treatment with a Triazole Inhibitor of the Mitochondrial Permeability Transition Pore Fully Corrects the Pathology of Sapje Zebrafish Lacking Dystrophin. Pharmacol. Res..

[58] Granato M., Eeden F.J.V., Schach U., Trowe T., Brand M., Furutani-Seiki M., Nüsslein-Volhard C. (1996). Genes Controlling and Mediating Locomotion Behavior of the Zebrafish Embryo and Larva. Development.

[59] Guyon J.R., Goswami J., Jun S.J., Thorne M., Howell M., Pusack T., Kunkel L.M. (2009). Genetic Isolation and Characterization of a Splicing Mutant of Zebrafish Dystrophin. Hum. Mol. Genet..

[60] Bassett D.I., Bryson-Richardson R.J., Daggett D.F., Gautier P., Keenan D.G., Currie P.D. (2003). Dystrophin Is Required for the Formation of Stable Muscle Attachments in the Zebrafish Embryo. Development.

[61] Berger J., Berger S., Hall T.E., Lieschke G.J., Currie P.D. (2010). Dystrophin-Deficient Zebrafish Feature Aspects of the Duchenne Muscular Dystrophy Pathology. J. Neuromuscul. Dis..

[62] Kawahara G., Kunkel L.M. (2013). Zebrafish Based Small Molecule Screens for Novel DMD Drugs. Drug Discov. Today Technol..

[63] Widrick J.J., Alexander M.S., Sanchez B., Gibbs D.E., Kawahara G., Beggs A.H., Kunkel L.M. (2016). Muscle Dysfunction in a Zebrafish Model of Duchenne Muscular Dystrophy. Physiol. Genom..

[64] Zulian A., Menazza S., Petronilli V., Argenton F., Merlini L., Sabatelli P., Bernardi P. (2017). Alisporivir Rescues Defective Mitochondrial Respiration in Duchenne Muscular Dystrophy. Pharmacol. Res..

[65] Widrick J.J., Kawahara G., Alexander M.S., Beggs A.H., Kunkel L.M. (2019). Discovery of Novel Therapeutics for Muscular Dystrophies Using Zebrafish Phenotypic Screens. J. Neuromuscul. Dis..

[66] Nesari V., Balakrishnan S., Nongthomba U. (2025). JAG1 Overexpression Partially Rescues Muscle Function in a Zebrafish Model of Duchenne Muscular Dystrophy. J. Genet..

[67] Lerma G., Ryhlick K.R., Carraher O.M., Beljan J.C., Amacher S.L. (2025). Validation of Duchenne Muscular Dystrophy Candidate Modifiers Using a CRISPR-Cas9-Based Approach in Zebrafish. bioRxiv.

[68] Nigro V., Savarese M. (2014). Genetic Basis of Limb-Girdle Muscular Dystrophies: The 2014 Update. Acta Myol..

[69] Straub V., Murphy A., Udd B., Corrado A., Aymé S., Bönneman C., Walter M. (2018). 229th ENMC International Workshop: Limb Girdle Muscular Dystrophies--Nomenclature and Reformed Classification Naarden, the Netherlands, 17--19 March 2017. J. Neuromuscul. Dis..

[70] Nigro V., Aurino S., Piluso G. (2011). Limb Girdle Muscular Dystrophies: Update on Genetic Diagnosis and Therapeutic Approaches. Curr. Opin. Neurol..

[71] Angelini C. (2020). LGMD. Identification, Description and Classification. Acta Myol..

[72] Georganopoulou D.G., Moisiadis V.G., Malik F.A., Mohajer A., Dashevsky T.M., Wuu S.T., Hu C.K. (2021). A Journey with LGMD: From Protein Abnormalities to Patient Impact. Protein J..

[73] Harms M.B., Sommerville R.B., Allred P., Bell S., Ma D., Cooper P., Baloh R.H. (2012). Exome Sequencing Reveals DNAJB6 Mutations in Dominantly-inherited Myopathy. Ann. Neurol..

[74] Sato T., Hayashi Y.K., Oya Y., Kondo T., Sugie K., Kaneda D., Nishino I. (2013). DNAJB6 Myopathy in an Asian Cohort and Cytoplasmic/Nuclear Inclusions. Neuromuscul. Disord..

[75] Sarparanta J., Jonson P.H., Golzio C., Sandell S., Luque H., Screen M., Udd B. (2012). Mutations Affecting the Cytoplasmic Functions of the Co-Chaperone DNAJB6 Cause Limb-Girdle Muscular Dystrophy. Nat. Genet..

[76] Nam T.S., Li W., Heo S.H., Lee K.H., Cho A., Shin J.H., Choi S.Y. (2015). A Novel Mutation in DNAJB6, p.(Phe91Leu), in Childhood-Onset LGMD1D with a Severe Phenotype. Neuromuscul. Disord..

[77] Melia M.J., Kubota A., Ortolano S., Vilchez J.J., Gamez J., Tanji K., Bonilla E., Palenzuela L., Fernandez-Cadenas I., Pristoupilova A. (2013). Limb-Girdle Muscular Dystrophy 1F Is Caused by a Microdeletion in the Transportin 3 Gene. Brain.

[78] Torella A., Fanin M., Mutarelli M., Peterle E., Del Vecchio Blanco F., Rispoli R., Savarese M., Garofalo A., Piluso G., Morandi L. (2013). Next-Generation Sequencing Identifies Transportin 3 as the Causative Gene for LGMD1F. PLoS ONE.

[79] Vieira N.M., Naslavsky M.S., Licinio L., Kok F., Schlesinger D., Vainzof M., Sanchez N., Kitajima J.P., Gal L., Cavacana N. (2014). A Defect in the RNA-Processing Protein HNRPDL Causes Limb-Girdle Muscular Dystrophy 1G (LGMD1G). Hum. Molec. Genet..

[80] Martinez-Thompson J.M., Niu Z., Tracy J.A., Moore S.A., Swenson A., Wieben E.D., Milone M. (2018). Autosomal dominant calpainopathy due to heterozygous CAPN3 c.643_663del21. Muscle Nerve.

[81] Vissing J., Barresi R., Witting N., Van Ghelue M., Gammelgaard L., Bindoff L.A., Straub V., Lochmuller H., Hudson J., Wahl C.M. (2016). A Heterozygous 21-Bp Deletion in CAPN3 Causes Dominantly Inherited Limb Girdle Muscular Dystrophy. Brain.

[82] Jobsis G.J., Keizers H., Vreijling J.P., de Visser M., Speer M.C., Wolterman R.A., Baas F., Bohlhuis P.A. (1996). Type VI Collagen Mutations in Bethlem Myopathy, an Autosomal Dominant Myopathy with Contractures. Nat. Genet..

[83] Tonelotto V., Consorti C., Facchinello N., Trapani V., Sabatelli P., Giraudo C., Spizzotin M., Cescon M., Bertolucci C., Bonaldo P. (2022). Collagen VI Ablation in Zebrafish Causes Neuromuscular Defects during Developmental and Adult Stages. Matrix Biol..

[84] Radev Z., Hermel J.M., Elipot Y., Bretaud S., Arnould S., Duchateau P., Ruggiero F., Joly J.S., Sohm F. (2015). A TALEN-Exon Skipping Design for a Bethlem Myopathy Model in Zebrafish. PLoS ONE.

[85] Telfer W.R., Busta A.S., Bonnemann C.G., Feldman E.L., Dowling J.J. (2010). Zebrafish Models of Collagen VI-Related Myopathies. Hum. Mol. Genet..

[86] Richard I., Broux O., Allamand V., Fougerousse F., Chiannilkulchai N., Bourg N., Brenguier L., Devaud C., Pasturaud P., Roudaut C. (1995). Mutations in the Proteolytic Enzyme Calpain 3 Cause Limb-Girdle Muscular Dystrophy Type 2A. Cell.

[87] Prykhozhij S.V., Caceres L., Ban K. (2023). Loss of Calpain3b in Zebrafish, a Model of Limb-Girdle Muscular Dystrophy, Increases Susceptibility to Muscle Defects Due to Elevated Muscle Activity. Genes.

[88] Chen F., Huang D., Shi H. (2020). Capn3 Depletion Causes Chk1 and Wee1 Accumulation and Disrupts Synchronization of Cell Cycle Reentry during Liver Regeneration after Partial Hepatectomy. Cell Regen.

[89] Bashir R., Britton S., Strachan T., Keers S., Vafiadaki E., Lako M., Richard I., Marchand S., Bourg N., Argov Z. (1998). A Gene Related to Caenorhabditis Elegans Spermatogenesis Factor Fer-1 Is Mutated in Limb-Girdle Muscular Dystrophy Type 2B. Nat. Genet..

[90] Kawahara G., Serafini P.R., Myers J.A., Alexander M.S., Kunkel L.M. (2011). Characterization of Zebrafish Dysferlin by Morpholino Knockdown. Biochem. Biophys. Res. Commun..

[91] Roostalu U., Strähle U. (2012). In Vivo Imaging of Molecular Interactions at Damaged Sarcolemma. Develop. Cell.

[92] Roberds S.L., Leturcq F., Allamand V., Piccolo F., Jeanpierre M., Anderson R.D., Lim L.E., Lee J.C., Tomé F.M., Romero N.B. (1994). Missense Mutations in the Adhalin Gene Linked to Autosomal Recessive Muscular Dystrophy. Cell.

[93] Romero N.B., Tome F.M.S., Leturcq F., El Kerch F., Azibi K., Bachner L., Anderson R.D., Roberds S.L., Campbell K.P., Fardeau M. (1994). Genetic Heterogeneity of Severe Childhood Autosomal Recessive Muscular Dystrophy with Adhalin (50 kDa Dystrophin-Associated Glycoprotein) Deficiency. C. R. Acad. Sci. III.

[94] Bönnemann C.G., Modi R., Noguchi S., Mizuno Y., Yoshida M., Gussoni E., McNally E.M., Duggan D.J., Angelini C., Hoffman E.P. (1995). β-Sarcoglycan (A3b) Mutations Cause Autosomal Recessive Muscular Dystrophy with Loss of the Sarcoglycan Complex. Nat. Genet..

[95] Lim L.E., Duclos F., Broux O., Bourg N., Sunada Y., Allamand V., Meyer J., Richard I., Moomaw C., Slaughter C. (1995). Beta-Sarcoglycan: Characterization and Role in Limb Girdle Muscular Dystrophy Linked to 4q12. Nat. Genet..

[96] Dalla Barba F., Soardi M., Mouhib L., Risato G., Akyürek E.E., Lucon-Xiccato T., Sandonà D. (2023). Modeling Sarcoglycanopathy in Danio Rerio. Int. J. Mol. Sci..

[97] Ben Othmane K., Ben Hamida M., Pericak-Vance M.A., Ben Hamida C., Blel S., Carter S.C., Bowcock A.M., Petruhkin K., Gilliam T.C., Roses A.D. (1992). Linkage of Tunisian Autosomal Recessive Duchenne-like Muscular Dystrophy to the Pericentromeric Region of Chromosome 13q. Nat. Genet..

[98] Noguchi S., MacNally E.M., Ben Othmane K., Hagiwara Y., Mizuno Y., Yoshida M., Yamamoto H., Bönneman C.G., Gussoni E., Denton P. (1995). Mutations in the Dystrophin-Associated Protein γ-Sarcoglycan in Chromosome 13 Muscular Dystrophy. Science.

[99] Nigro V., De Sa’ Moreira E., Piluso G., Vainzof M., Belsito A., Politano L. (1996). Autosomal Recessive Limb- Girdle Muscular Dystrophy, LGMD 2F, Is Caused by a Mutation in the Sarcoglycan Gene. Nat. Genet..

[100] Cheng L., Guo X.F., Yang X.Y., Chong M., Cheng J., Li G., Lu D.R. (2006). δ-Sarcoglycan Is Necessary for Early Heart and Muscle Development in Zebrafish. Biochem. Biophys. Res. Commun..

[101] Guyon J.R., Mosley A.N., Jun S.J., Montanaro F., Steffen L.S., Zhou Y., Kunkel L.M. (2005). δ-Sarcoglycan Is Required for Early Zebrafish Muscle Organization. Exp. Cell Res..

[102] Moreira E.S., Vainzof M., Marie S.K., Sertie A.L., Zatz M., Passos-Bueno M.R. (1997). The Seventh Form of Autosomal Recessive Limb-Girdle Muscular Dystrophy Is Mapped to 17q11-12. Am. J. Hum. Genet..

[103] Moreira E.S., Wiltshire T.J., Faulkner G., Nilforoushan A., Vainzof M., Suzuki O.T., Valle G., Reeves R., Zatz M., Passos-Bueno M.R. (2000). Limb-Girdle Muscular Dystrophy Type 2G Is Caused by Mutations in the Gene Encoding the Sarcomeric Protein Telethonin. Nat. Genet..

[104] Zhang R., Yang J., Zhu J., Xu X. (2009). Depletion of Zebrafish Tcap Leads to Muscular Dystrophy via Disrupting Sarcomere-Membrane Interaction, Not Sarcomere Assembly. Hum. Molec. Genet..

[105] Frosk P., Weiler T., Nylen E., Sudha T., Greenberg C.R., Morgan K., Fujiwara T.M., Wrogemann K. (2002). Limb-Girdle Muscular Dystrophy Type 2H Associated with Mutation in TRIM32, a Putative E3-Ubiquitin-Ligase Gene. Am. J. Hum. Genet..

[106] Brockington M., Blake D.J., Prandini P., Brown S.C., Torelli S., Benson M.A., Ponting C.P., Estournet B., Romero N.B., Mercuri E. (2001). Mutations in the Fukutin-Related Protein Gene (*FKRP*) Cause a Form of Congenital Muscular Dystrophy with Secondary Laminin A2 Deficiency and Abnormal Glycosylation of α-Dystroglycan. Am. J. Hum. Genet..

[107] Serafini P.R., Feyder M.J., Hightower R.M., Garcia-Perez D., Vieira N.M., Lek A., Gibbs D.E., Moukha-Chafiq O., Augelli-Szafran C.E., Kawahara G. (2018). A Limb-Girdle Muscular Dystrophy 2I Model of Muscular Dystrophy Identifies Corrective Drug Compounds for Dystroglycanopathies. JCI Insight.

[108] Wood A.J., Lin C.H., Li M., Nishtala K., Alaei S., Rossello F., Sonntag C., Hersey L., Miles L.B., Krisp C. (2021). FKRP-Dependent Glycosylation of Fibronectin Regulates Muscle Pathology in Muscular Dystrophy. Nat. Commun..

[109] Lin Y.Y., White R.J., Torelli S., Cirak S., Muntoni F., Stemple D.L. (2011). Zebrafish Fukutin Family Proteins Link the Unfolded Protein Response with Dystroglycanopathies. Hum. Mol. Genet..

[110] Wood A.J., Muller J.S., Jepson C.D., Laval S.H., Lochmuller H., Bushby K., Barresi R., Straub V. (2011). Abnormal Vascular Development in Zebrafish Models for Fukutin and FKRP Deficiency. Hum. Mol. Genet..

[111] Thornhill P., Bassett D., Lochmuller H., Bushby K., Straub V. (2008). Developmental Defects in a Zebrafish Model for Muscular Dystrophies Associated with the Loss of Fukutin-Related Protein (FKRP). Brain.

[112] Kawahara G., Guyon J.R., Nakamura Y., Kunkel L.M. (2010). Zebrafish Models for Human FKRP Muscular Dystrophies. Hum. Mol. Genet..

[113] Udd B., Rapola J., Nokelainen P., Arikawa E., Somer H. (1992). Nonvacuolar Myopathy in a Large Family with Both Late Adult Onset Distal Myopathy and Severe Proximal Muscular Dystrophy. J. Neurol. Sci..

[114] Hackman P., Vihola A., Haravuori H., Marchand S., Sarparanta J., de Seze J., Labeit S., Witt C., Peltonen L., Richard I. (2002). Tibial Muscular Dystrophy Is a Titinopathy Caused by Mutations in TTN, the Gene Encoding the Giant Skeletal-Muscle Protein Titin. Am. J. Hum. Genet..

[115] Zou J., Tran D., Baalbaki M., Tang L.F., Poon A., Pelonero A., Titus E.W., Yuan C., Shi C., Patchava S. (2015). An Internal Promoter Underlies the Difference in Disease Severity between N- and C-Terminal Truncation Mutations of Titin in Zebrafish. eLife.

[116] Steffen L.S., Guyon J.R., Vogel E.D., Howell M.H., Zhou Y., Weber G.J., Zon L.I., Kunkel L.M. (2007). The Zebrafish Runzel Muscular Dystrophy Is Linked to the Titin Gene. Dev. Biol..

[117] Dincer P., Balci B., Yuva Y., Talim B., Brockington M., Dincel D., Torelli S., Brown S., Kale G., Haliloglu G. (2003). A Novel Form of Recessive Limb Girdle Muscular Dystrophy with Mental Retardation and Abnormal Expression of Alpha-Dystroglycan. Neuromusc. Disord..

[118] Balci B., Uyanik G., Dincer P., Gross C., Willer T., Talim B., Haliloglu G., Kale G., Hehr U., Winkler J. (2005). An Autosomal Recessive Limb Girdle Muscular Dystrophy (LGMD2) with Mild Mental Retardation Is Allelic to Walker-Warburg Syndrome (WWS) Caused by a Mutation in the POMT1 Gene. Neuromusc. Disord..

[119] Avsar-Ban E., Ishikawa H., Manya H., Watanabe M., Akiyama S., Miyake H., Endo T., Tamaru Y. (2010). Protein O-Mannosylation Is Necessary for Normal Embryonic Development in Zebrafish. Glycobiology.

[120] Bolduc V., Marlow G., Boycott K.M., Saleki K., Inoue H., Kroon J., Itakura M., Robitaille Y., Parent L., Baas F. (2010). Recessive Mutations in the Putative Calcium-Activated Chloride Channel Anoctamin 5 Cause Proximal LGMD2L and Distal MMD3 Muscular Dystrophies. Am. J. Hum. Genet..

[121] Godfrey C., Escolar D., Brockington M., Clement E.M., Mein R., Jimenez-Mallebrera C., Torelli S., Feng L., Brown S.C., Sewry C.A. (2006). Fukutin gene mutations in steroid-responsive limb girdle muscular dystrophy. Ann. Neurol..

[122] Godfrey C., Clement E., Mein R., Brockington M., Smith J., Talim B., Straub V., Robb S., Quinlivan R., Feng L. (2007). Refining Genotype-Phenotype Correlations in Muscular Dystrophies with Defective Glycosylation of Dystroglycan. Brain.

[123] Clement E.M., Godfrey C., Tan J., Brockington M., Torelli S., Feng L., Brown S.C., Jimenez-Mallebrera C., Sewry C.A., Longman C. (2008). Mild POMGnT1 Mutations Underlie a Novel Limb-Girdle Muscular Dystrophy Variant. Arch. Neurol..

[124] Raducu M., Baets J., Fano O., Van Coster R., Cruces J. (2012). Promoter alteration causes transcriptional repression of the POMGNT1 gene in limb-girdle muscular dystrophy type 2O. Europ. J. Hum. Genet..

[125] Hara Y., Balci-Hayta B., Yoshida-Moriguchi T., Kanagawa M., Beltran-Valero de Bernabe D., Gundesli H., Willer T., Satz J.S., Crawford R.W., Burden S.J. (2011). A Dystroglycan Mutation Associated with Limb-Girdle Muscular Dystrophy. New Eng. J. Med..

[126] Parsons M.J., Campos I., Hirst E.M., Stemple D.L. (2002). Removal of Dystroglycan Causes Severe Muscular Dystrophy in Zebrafish Embryos. Development.

[127] Gupta V., Kawahara G., Gundry S.R., Chen A.T., Lencer W.I., Zhou Y., Zon L.I., Kunkel L.M., Beggs A.H. (2011). The Zebrafish Dag1 Mutant: A Novel Genetic Model for Dystroglycanopathies. Hum. Mol. Genet..

[128] Gundesli H., Talim B., Korkusuz P., Balci-Hayta B., Cirak S., Akarsu N.A., Topaloglu H., Dincer P. (2010). Mutation in Exon 1f of PLEC, Leading to Disruption of Plectin Isoform 1f, Causes Autosomal-Recessive Limb-Girdle Muscular Dystrophy. Am. J. Hum. Genet..

[129] Bogershausen N., Shahrzad N., Chong J.X., von Kleist-Retzow J.-C., Stanga D., Li Y., Bernier F.P., Loucks C.M., Wirth R., Puffenberger E.G. (2013). Recessive TRAPPC11 Mutations Cause a Disease Spectrum of Limb Girdle Muscular Dystrophy and Myopathy with Movement Disorder and Intellectual Disability. Am. J. Hum. Genet..

[130] Ulhaq Z.S., Ogino Y., Tse W.K.F. (2023). FGF8 Rescues Motor Deficits in Zebrafish Model of Limb-Girdle Muscular Dystrophy R18. Biochem. Biophys. Res. Commun..

[131] Carss K.J., Stevens E., Foley A.R., Cirak S., Riemersma M., Torelli S., Hoischen A., Willer T., van Scherpenzeel M., Moore S.A. (2013). Mutations in GDP-Mannose Pyrophosphorylase B Cause Congenital and Limb-Girdle Muscular Dystrophies Associated with Hypoglycosylation of Alpha-Dystroglycan. Am. J. Hum. Genet..

[132] Tasca G., Moro F., Aiello C., Cassandrini D., Fiorillo C., Bertini E., Bruno C., Santorelli F.M., Ricci E. (2013). Limb-Girdle Muscular Dystrophy with Alpha-Dystroglycan Deficiency and Mutations in the ISPD Gene. Neurology.

[133] Servian-Morilla E., Takeuchi H., Lee T.V., Clarimon J., Mavillard F., Area-Gomez E., Rivas E., Nieto-Gonzalez J.L., Rivero M.C., Cabrera-Serrano M. (2016). A POGLUT1 Mutation Causes a Muscular Dystrophy with Reduced Notch Signaling and Satellite Cell Loss. EMBO Molec. Med..

[134] Gualandi F., Urciuolo A., Martoni E., Sabatelli P., Squarzoni S., Bovolenta M., Messina S., Mercuri E., Franchella A., Ferlini A. (2009). Autosomal Recessive Bethlem Myopathy. Neurology.

[135] Gavassini B.F., Carboni N., Nielsen J.E., Danielsen E.R., Thomsen C., Svenstrup K., Bello L., Maioli M.A., Marrosu G., Ticca A.F. (2011). Clinical and Molecular Characterization of Limb-Girdle Muscular Dystrophy Due to LAMA2 Mutations. Muscle Nerve.

[136] Hall T.E., Bryson-Richardson R.J., Berger S., Jacoby A.S., Cole N.J., Hollway G.E., Berger J., Currie P.D. (2007). The Zebrafish Candyfloss Mutant Implicates Extracellular Matrix Adhesion Failure in Laminin Alpha2-Deficient Congenital Muscular Dystrophy. Proc. Natl. Acad. Sci. USA.

[137] Gupta V.A., Kawahara G., Myers J.A., Chen A.T., Hall T.E., Manzini M.C., Currie P.D., Zhou Y., Zon L.I., Kunkel L.M. (2012). A Splice Site Mutation in Laminin-Alpha2 Results in a Severe Muscular Dystrophy and Growth Abnormalities in Zebrafish. PLoS ONE.

[138] Endo Y., Dong M., Noguchi S., Ogawa M., Hayashi Y.K., Kuru S., Sugiyama K., Nagai S., Ozasa S., Nonaka I. (2015). Milder Forms of Muscular Dystrophy Associated with POMGNT2 Mutations. Neurol. Genet..

[139] Schindler R.F.R., Scotton C., Zhang J., Passarelli C., Ortiz-Bonnin B., Simrick S., Schwerte T., Poon K.-L., Fang M., Rinne S. (2016). POPDC1-S201F Causes Muscular Dystrophy and Arrhythmia by Affecting Protein Trafficking. J. Clin. Investig..

[140] Vissing J., Johnson K., Topf A., Nafissi S., Diaz-Manera J., French V.M., Schindler R.F., Sarathchandra P., Lokken N., Rinne S. (2019). POPDC3 Gene Variants Associate with a New Form of Limb Girdle Muscular Dystrophy. Ann. Neurol..

[141] Coppens S., Barnard A.M., Puusepp S., Pajusalu S., Ounap K., Vargas-Franco D., Bruels C.C., Donkervoort S., Pais L., Chao K.R. (2021). A Form of Muscular Dystrophy Associated with Pathogenic Variants in JAG2. Am. J. Hum. Genet..

[142] Yogev Y., Shorer Z., Koifman A., Wormser O., Drabkin M., Halperin D., Dolgin V., Proskorovski-Ohayon R., Hadar N., Davidov G. (2023). Limb Girdle Muscular Disease Caused by HMGCR Mutation and Statin Myopathy Treatable with Mevalonolactone. Proc. Nat. Acad. Sci. USA.

[143] Bouchard C., Tremblay J.P. (2023). Limb–Girdle Muscular Dystrophies Classification and Therapies. J. Clin. Med..

[144] Zelikovich A.S., Joslin B.C., Casey P., McNally E.M., Ajroud-Driss S. (2022). An Open Label Exploratory Clinical Trial Evaluating Safety and Tolerability of Once-Weekly Prednisone in Becker and Limb-Girdle Muscular Dystrophy. J. Neuromuscul. Dis..

[145] Ganaraja V.H., Polavarapu K., Bardhan M., Preethish-Kumar V., Leena S., Anjanappa R.M., Vengalil S., Nashi S., Arunachal G., Gunasekaran S. (2021). Disease Progression and Mutation Pattern in a Large Cohort of LGMD R1/LGMD 2A Patients from India. Glob Med. Genet..

[146] Angelini C. (2025). Treatabolome for Finely Targeting Muscle Pathology in LGMD. Acta Myol..

[147] Bardakov S.N., Sorochanu I., Mkrtchyan L.A. (2025). Calpainopathy (Limb-Girdle Muscular Dystrophy Type R1): Clinical Features, Diagnostic Approaches, and Biotechnological Treatment Methods. J. Neuromuscul. Dis..

[148] Anwar S., Yokota T. (2024). The Dysferlinopathies Conundrum: Clinical Spectra, Disease Mechanism and Genetic Approaches for Treatments. Biomolecules.

[149] Carotti M., Fecchio C., Sandonà D. (2017). Emerging Therapeutic Strategies for Sarcoglycanopathy. Expert Opin. Orphan Drugs.

[150] Scano M., Benetollo A., Dalla Barba F., Sandonà D. (2024). Advanced Therapeutic Approaches in Sarcoglycanopathies. Curr. Opin. Pharmacol..

[151] Nigro V. (2003). Molecular Bases of Autosomal Recessive Limb-Girdle Muscular Dystrophies. Acta Myol..

[152] Fanin M., Nascimbeni A.C., Aurino S., Tasca E., Pegoraro E., Nigro V., Angelini C. (2009). Frequency of LGMD Gene Mutations in Italian Patients with Distinct Clinical Phenotypes. J. Neurol..

[153] Gaina G., Popa A. (2021). Muscular Dystrophy: Experimental Animal Models and Therapeutic Approaches. Exp. Ther. Med..

[154] Mendell J.R., Pozsgai E.R., Lewis S., Griffin D.A., Lowes L.P., Alfano L.N., Lehman K.J., Church K., Reash N.F., Iammarino M.A. (2024). Gene Therapy with Bidridistrogene Xeboparvovec for Limb-Girdle Muscular Dystrophy Type 2E/R4: Phase 1/2 Trial Results. Nat. Med..

[155] Sharma A., Sane H., Gokulchandran N., Gandhi S., Bhovad P., Khopkar D., Paranjape A., Bhagwanani K., Badhe P. (2015). The Role of Cell Transplantation in Modifying the Course of Limb Girdle Muscular Dystrophy: A Longitudinal 5-Year Study. Degener. Neurol. Neuromuscul. Dis..

[156] Carotti M., Scano M., Fancello I., Richard I., Risato G., Bensalah M., Soardi M., Sandonà D. (2020). Combined Use of CFTR Correctors in LGMD2D Myotubes Improves Sarcoglycan Complex Recovery. Int. J. Mol. Sci..

[157] Scano M., Benetollo A., Nogara L., Bondì M., Dalla Barba F., Soardi M., Furlan S., Akyurek E.E., Caccin P., Carotti M. (2022). CFTR Corrector C17 Is Effective in Muscular Dystrophy, in Vivo Proof of Concept in LGMDR3. Hum. Mol. Genet..

[158] Benetollo A., Parrasia S., Scano M., Biasutto L., Rossa A., Nogara L., Blaauw B., Dalla Barba F., Caccin P., Carotti M. (2025). The Novel Use of the CFTR Corrector C17 in Muscular Dystrophy: Pharmacological Profile and in Vivo Efficacy. Biochem. Pharmacol..

[159] Odermatt A., Taschner P.E.M., Khanna V.K., Busch H.F., Karpati G., Jablecki C.K. (1996). Mutations in the Gene—Encoding SERCA1, the Fast--Twitch Skeletal Muscle Sarcoplasmic Reticulum Ca2+ ATPase, Are Associated with Brody Disease. Nat. Genet..

[160] Brody I.A. (1996). Muscle Contracture Induced by Exercise: A Syndrome Attributable to Decreased Relaxing Factor. New Eng. J. Med..

[161] Karpati G., Charuk J., Carpenter S., Jablecki C., Holland P. (1986). Myopathy Caused by a Deficiency of Ca2+-Adenosine Triphosphatase in Sarcoplasmic Reticulum (Brody’s Disease). Ann. Neurol..

[162] Taylor D.J., Brosnan M.J., Arnold D.L., Bore P.J., Styles P., Walton J., Radda G.K. (1988). Ca^2+^-ATPase Deficiency in a Patient with an Exertional Muscle Pain Syndrome. J. Neurol. Neurosurg. Psychiatry.

[163] Wevers R.A., Poels P.J., Joosten E.M., Steenbergen G.G., Benders A.A., Veerkamp J.H. (1992). Ischaemic Forearm Testing in a Patient with Ca^2+^-ATPase Deficiency. J. Inherit. Metab. Dis..

[164] Poels P.J., Wevers R.A., Braakhekke J.P., Benders A.A., Veerkamp J.H., Joosten E.M. (1993). Exertional Rhabdomyolysis in a Patient with Calcium Adenosine Triphosphatase Deficiency. J. Neurol. Neurosurg. Psychiatry..

[165] Benders A.A., Veerkamp J.H., Oosterhof A., Jongen P.J., Bindels R.J., Smitn L.M. (1994). Ca^2+^ Homeostasis in Brody’s Disease. A Study in Skeletal Muscle and Cultured Muscle Cells and the Effects of Dantrolene an Verapamil. J. Clin. Investig..

[166] Guglielmi V., Vattemi G., Gualandi F., Voermans N.C., Marini M., Scotton C., Tomelleri G. (2013). SERCA1 Protein Expression in Muscle of Patients with Brody Disease and Brody Syndrome and in Cultured Human Muscle Fibers. Mol. Genet. Metab..

[167] Pan Y., Zvaritch E., Tupling A.R., Rice W.J., de Leon S., Rudnicki M., McKerlie C., Banwell B.L., MacLennan D.H. (2003). Targeted Disruption of the ATP2A1 Gene Encoding the Sarco(Endo)Plasmic Reticulum Ca^2+^ ATPase Isoform 1 (SERCA1) Impairs Diaphragm Function and Is Lethal in Neonatal Mice. J. Biol. Chem..

[168] Sacchetto R., Testoni S., Gentile A., Damiano E., Rossi M., Liguori R., Drögemüller C., Mascarello F. (2009). A Defective SERCA1 Protein Is Responsible for Congenital Pseudomyotonia in Chianina Cattle. Am. J. Pathol..

[169] Bianchini E., Testoni S., Gentile A., Calì T., Ottolini D., Villa A., Brini M., Betto R., Mascarello F., Nissen P. (2014). Inhibition of Ubiquitin Proteasome System Rescues the Defective Sarco(Endo)Plasmic Reticulum Ca^2+^-ATPase (SERCA1. Protein Causing Chianina Cattle Pseudomyotonia. J. Biol. Chem..

[170] Akyürek E.E., Busato F., Murgiano L., Bianchini E., Carotti M., Sandonà D., Sacchetto R. (2022). Differential Analysis of Gly211Val and Gly286Val Mutations Affecting Sarco (Endo) Plasmic Reticulum Ca^2+^-ATPase (SERCA1) in Congenital Pseudomyotonia Romagnola Cattle. Int. J. Mol. Sci..

[171] Hirata H., Saint-Amant L., Waterbury J., Cui W., Zhou W., Li Q., Goldman D., Granato M., Kuwada J.Y. (2004). Accordion, a Zebrafish Behavioral Mutant, Has a Muscle Relaxation Defect Due to a Mutation in the ATPase Ca^2+^ Pump SERCA1. Development.

[172] Gleason M.R., Armisen R., Verdecia M.A., Sirotkin H., Brehm P., Mandel G. (2004). A Mutation in Serca Underlies Motility Dysfunction in Accordion Zebrafish. Dev. Biol..

[173] Toyoshima C., Inesi G. (2004). Structural Basis of Ion Pumping by Ca^2+^-ATPase of the Sarcoplasmic Reticulum. Annu. Rev. Biochem..

[174] Akyürek E.E., Greco F., Tesoriero C., Dalla Barba F., Carotti M., Gorni G., Sacchetto R. (2024). The Accordion Zebrafish Tq206 Mutant in the Assessment of a Novel Pharmaceutical Approach to Brody Myopathy. Int. J. Mol. Sci..

[175] Bédard P., Gauvin S., Ferland K., Caneparo C., Pellerin È., Chabaud S., Bolduc S. (2020). Innovative human three-dimensional tissue-engineered models as an alternative to animal testing. Bioengineering.

[176] Veinotte C.J., Dellaire G., Berman J.N. (2014). Hooking the big one: The potential of zebrafish xenotransplantation to reform cancer drug screening in the genomic era. Dis. Models Mech..

[177] Bartoli M., Gicquel E., Barrault L., Soheili T., Malissen M., Malissen B., Vincent-Lacaze N., Perez N., Udd B., Danos O. (2008). Mannosidase I inhibition rescues the human alpha-sarcoglycan R77C recurrent mutation. Hum. Mol. Genet..

[178] Kobuke K., Piccolo F., Garringer K.W., Moore S.A., Sweezer E., Yang B., Campbell K.P. (2008). A common disease-associated missense mutation in alpha-sarcoglycan fails to cause muscular dystrophy in mice. Hum. Mol. Genet..

[179] Henriques S.F., Patissier C., Bourg N., Fecchio C., Sandona D., Marsolier J., Richard I. (2018). Different outcome of sarcoglycan missense mutation between human and mouse. PLoS ONE.

[180] Lieschke G.J., Currie P.D. (2007). Animal models of human disease: Zebrafish swim into view. Nat. Rev. Genet..

[181] Strähle U., Scholz S., Geisler R., Greiner P.L., Hollert H., Rastegar S., Schumacher A., Selderslaghs I., Weiss C., Witters H. (2012). Zebrafish embryos as an alternative to animal experiments—A commentary on the definition of the onset of protected life stages in animal welfare regulations. Reprod. Toxicol..

[182] Cassar S., Adatto I., Freeman J.L., Gamse J.T., Iturria I., Lawrence C., Muriana A., Peterson R.T., Van Cruchten S., Zon L.I. (2020). Use of Zebrafish in Drug Discovery Toxicology. Chem. Res. Toxicol..

[183] Directive E. (2010). 63/EU of the European Parliament and of the Council of 22 September 2010 on the protection of animals used for scientific purposes. Off. J. Eur. Union.

[184] MacArthur Clark J. (2018). The 3Rs in research: A contemporary approach to replacement, reduction and refinement. Br. J. Nutr..

[185] Bailone R.L., Fukushima H.C.S., Ventura Fernandes B.H., De Aguiar L.K., Corrêa T., Janke H., Borra R.C. (2020). Zebrafish as an Alternative Animal Model in Human and Animal Vaccination Research. Lab. Anim. Res..

